# Role of PET and SPECT in the Study of Amyotrophic Lateral Sclerosis

**DOI:** 10.1155/2014/237437

**Published:** 2014-04-10

**Authors:** Angelina Cistaro, Vincenzo Cuccurullo, Natale Quartuccio, Marco Pagani, Maria Consuelo Valentini, Luigi Mansi

**Affiliations:** ^1^Positron Emission Tomography Centre IRMET S.p.A., Euromedic Inc., V. O. Vigliani 89/A, 10136 Turin, Italy; ^2^PET Pediatric AIMN InterGroup, V. O. Vigliani 89/A, 10136 Turin, Italy; ^3^Institute of Cognitive Sciences and Technologies, National Research Council, Piazzale Aldo Moro 7, 00185 Rome, Italy; ^4^Nuclear Medicine Unit, Department of Clinical and Experimental Internistic “F. Magrassi, A. Lanzara”, Seconda Università di Napoli, Vico Luigi de Crecchio 16, 80138 Naples, Italy; ^5^Nuclear Medicine Unit, Department of Biomedical Sciences and of Morphological and Functional Images, University of Messina, Via Consolare Valeria 1, 98125 Messina, Italy; ^6^Department of Nuclear Medicine, Karolinska University Hospital, Solnavägen 1, Solna, 171 77 Stockholm, Sweden; ^7^Department of Neuroradiology, San Giovanni Battista-OIRM-S.Anna-CTO Hospital, Via Cherasco 15, 10126 Turin, Italy

## Abstract

Amyotrophic lateral sclerosis has been defined as a “heterogeneous group of neurodegenerative syndromes characterized by progressive muscle paralysis caused by the degeneration of motor neurons allocated in primary motor cortex, brainstem, and spinal cord.” A comprehensive diagnostic workup for ALS usually includes several electrodiagnostic, clinical laboratory and genetic tests. Neuroimaging exams, such as computed tomography, magnetic resonance imaging and spinal cord myelogram, may also be required. Nuclear medicine, with PET and SPECT, may also play a role in the evaluation of patients with ALS, and provide additional information to the clinicians. This paper aims to offer to the reader a comprehensive review of the different radiotracers for the assessment of the metabolism of glucose (FDG), the measurement of cerebral blood flow (CBF), or the evaluation of neurotransmitters, astrocytes, and microglia by means of newer and not yet clinically diffuse radiopharmaceuticals.

## 1. Nuclear Medicine as Functional and/or Molecular Imaging in the Study of Nervous System

The living being is composed of biomolecules in dynamic equilibrium between them in the definition of the so-called homeostasis, which represents the physiology [1]. The disease can be considered the alteration of this system, being the representation of an imbalance which is expressed initially as functional impairment, sometimes reversible [2]. However, beside the possibility of a return to normal condition, there is the risk of further evolution towards an alteration that may become apparent at the morphostructural level [3].

There is therefore a gradation in the progress of the disease that can be identified according to a timeline that shows the morphostructural modification as a late event, preceded by functional alteration [4]. Consequently, functional imaging, that is, the representation of pathophysiological alterations, may be more precocious in the early detection of disease with respect to a diagnostic imaging based on morphostructural premises [5]. Furthermore functional imaging has a greater capacity in assessing the prognosis and the relationship with the therapy in the individual patient, being pathophysiological changes a better predictor of the evolution of the disease and/or the effectiveness of therapeutic action [6].

In diagnostic imaging even more interesting is the possibility of studying the molecular mechanisms that underlie the disease, allowing the representation of the initial pathological alteration [7]. Without dwelling on technical insights, there are two basic systems of study: Single Photon Emission Tomography (SPECT), which creates images using single gamma radiations emitted by gamma-emitting radionuclides, and Positron Emission Tomography (PET), which displays images resulting from double gamma photons in coincidence, which derive from annihilation of positrons [5]. Both devices may be integrated within the so-called hybrid machines, which have made possible a technological revolution that has given and continues to bear fruits even in the clinical setting [8]. Recently another promising hybrid device, PET/MRI, was developed. PET/MRI represents an exciting novel imaging option for oncological as well as neurological applications [9].

In nuclear medicine, it is possible to obtain a large number of radioactive probes and then “trace” multiple functions and molecular mechanisms in the body [7]. Through them, it is possible to acquire sensitive and precise information, which creates the basis for investigating the earliest levels of disease, resulting in favourable therapeutic implications [5]. Though the primary role in clinical diagnosis, the functional information has however to be integrated by the morphostructural one [10]. In this way, it is at first possible to increase the diagnostic accuracy, better understanding and delineating the limits of normal and pathological anatomical structures, with a significant improvement either in sensitivity and specificity [11]. Furthermore, because of their better spatial resolution and/or of the different presuppositions underlying the image, morphostructural techniques, such as CT, MRI, and US, may also detect abnormalities that are not visible with a functional study [11].

The clinical analysis of neurological diseases with nuclear medicine is at the present connected with three main categories of radiotracers studying (1) perfusion, (2) metabolism, and (3) receptors [5].

Among all used radiotracers, the most important is still the first among those proposed, namely, the F-18 fluoro-deoxyglucose (FDG), which traces the metabolism of glucose. Currently, being mainly used for oncologic applications [12, 13], PET-FDG has a clinical interest also in nonneoplastic pathologies, in particular in inflammatory diseases [14]. Furthermore, FDG plays an important role also in the evaluation of neurological diseases, first of all in dementia [15].

Among the most interesting applications of this method in the brain, there is certainly that, related to Amyotrophic Lateral Sclerosis (ALS), which is the subject of this paper. Together with FDG, further information in ALS may be acquired using radiotracers measuring cerebral blood flow (CBF) or newer and not yet clinically diffuse radiopharmaceuticals, as those allowing the evaluation of neurotransmitters, astrocytes, and microglia [16].

## 2. Amyotrophic Lateral Sclerosis

Amyotrophic Lateral Sclerosis (ALS), also known as Charcot, Lou Gehrig, or motor neuron's disease, has been defined as a “heterogeneous group of neurodegenerative syndromes characterized by progressive muscle paralysis caused by the degeneration of motor neurons allocated in primary motor cortex, brainstem, and spinal cord” [17]. The disease was identified in 1874 by JM Charcot as “a degenerative process involving neurons in the anterior horns of the spinal cord and in motor nuclei of brainstem” [18]. ALS affects not only motor neurons but also their nonneural neighbours, including astrocytes and microglia, whose involvement amplifies the initial damage and drives disease progression and spread [19]. The main clinical variants of ALS include Primary Muscular Atrophy (PMA), Primary Lateral Sclerosis (PLS), and Progressive Bulbar Palsy (PBP) [19].

Being currently still unknown aetiology, many etiopathogenetic hypotheses have been made for ALS. Mutations in several genes have been demonstrated to be linked to ALS, Cu/Zn superoxide dismutase (SOD1), TAR DNA-binding protein (TARDBP), the gene encoding the TAR DNA-binding protein 43—TDP-43, the fused in sarcoma/translocated in liposarcoma (FUS/TLS) protein, and chromosome 9 open reading frame 72 gene (C9ORF72) being the most important [20]. Other mutated genes involved in ALS are PFN1, OPTN, VCP, UBQLN2, ANG, FIG4, DCTN1, and CHMP2B [20]. Furthermore ALS has been related to (1) effect of exotoxins, which would lead to an excessive stimulation of glutaminergic postsynaptic NMDA and AMPA receptors; (2) oxidative stress, with accumulation of reactive oxygen; (3) mitochondrial dysfunction, with morphological and biochemical abnormalities. Among the factors involved are also included the alteration of the axonal transport, the deposit of aggregates of proteic neurofilaments, the dysfunction of glial cells, and the deficit of neurotropic factors [19].

With regard to the possibility of a role of genetic factors, it should be remembered that the disease is expressed in 90% of cases as sporadic form, being identified as a familiar disease, with autosomal dominant inheritance, in less than 10% of patients. Being a little more frequent in males (M/F = 1.3), ALS has an average age of onset at 55–65 years. The incidence is 1.5–2.7 and the prevalence is 2.7–7.4 per 100,000 inhabitants/year. Progression and severity can vary greatly from one patient to another. The mortality rate is 1.54–2.55 per 100,000 patients, with a median survival of 3–5 years. The main cause of death is the failure of the respiratory muscles [21].

## 3. Diagnostic Workup of ALS

ALS is a very difficult disease to diagnose and at the present there is no test or procedure to confirm without any doubt the final diagnosis [22]. More frequently, clinical suspicion emerges through a careful clinical examination, repeated over time by an expert neurologist, and a series of diagnostic tests to rule out other possible disorders justifying clinical symptoms. According to ALS Association (fighting Lou Gehrig's disease) a comprehensive diagnostic workup has to include most, if not all, of the following procedures [21]:

- electrodiagnostic tests including conventional electromyography (EMG), nerve conduction studies, transcranial magnetic stimulation, central motor conduction studies, and quantitative electromyography
- neuroimaging including computed tomography (CT) scanning or magnetic resonance imaging (MRI) of the brain and spinal cord myelogram of cervical spine,
- clinical laboratory tests which will include muscle enzymes (serum creatine kinase (unusual above ten times upper limit of normal), ALT, AST, and LDH), serum creatinine (related to loss of skeletal muscle mass), hypochloremia, increased bicarbonate (related to advanced respiratory compromise), and elevated CSF protein (uncommonly more than 100 mg/dL)
- muscle and/or nerve biopsy,
- genetic testing.

The clinical role of diagnostic imaging, mainly interpreted by MRI, is mainly individuated in excluding alternative diseases, being nontypical and/or difficult to be detected diagnostic signs univocally defining ALS. Being this disease strictly connected with the motor neuron, the pathological damage should be evidenced exclusively at the level of the motor cortex and/or at the level of the subcortical structures involved in the motor system [21].

The currently most used diagnostic criteria for ALS are those of El Escorial World Federation of Neurology [23], which divide ALS into 5 diagnostic categories: clinically definite ALS, clinically probable ALS, clinically probable-laboratory supported ALS, and clinically possible ALS (Table 1).

## 4. Glucose Metabolism and Cerebral Blood Flow in ALS Patients

Either glucose metabolism or cerebral blood flow is similarly reduced in patients with ALS [24–29]. Surprisingly, since first studies with FDG PET performed in early 80's [30] in patients with upper motor neuron signs compared to age-matched control subjects, a decreased activity was observed not only in the motor primary and accessory medial motor cortex, but also at level of parietal and occipital lobes, being spared visual areas.

A frontal lobe dysfunction was also demonstrated in nondemented ALS patients by Abrahams et al. [31], measuring cerebral blood flow (rCBF) with PET. The study was based on an activation paradigm of executive frontal lobe function (verbal fluency), which contrasted with rCBF during word generation and word repetition. A PET scan was performed in groups of age matched individuals constituted by patients with ALS, respectively, affected (ALSi = impaired) or not (ALSu = unimpaired) with a cognitive impairment, both compared with healthy controls. The ALSi subjects displayed significantly impaired activation in cortical and subcortical regions including the dorsolateral prefrontal cortex, lateral premotor cortex, medial prefrontal and premotor cortices, and insular cortex bilaterally and the anterior thalamic nuclear complex. Although the three groups showed matched word generation performance on the scanning paradigm, the ALSu group displayed a relatively unimpaired pattern of activation. These results are in agreement with an extramotor neuronal involvement in some non-demented ALS patients that develops probably along a thalamo-frontal association pathway.

Dalakas et al. [24], using FDG PET, observed that in patients with upper motor neuron signs, the mean cortical Regional Cerebral Metabolic Rate of Glucose Consumption (rCMRGlc) was significantly lower than in normal subjects. Moreover, subsequent reduction in the rCMRGlc, in agreement with the clinical worsening, was observed in 3 out of the 4 patients who underwent repeated PET scan. Conversely, a normal or near-normal rCMRGlc was seen throughout the brain in ALS patients with disease confined to lower motor neurons and in 3 subjects with lower motor neuron disease, depending from old paralytic poliomyelitis. As already observed in cerebellar diaschisis [32], these data demonstrate that a hypometabolism may be seen in a structurally normal cortex, in case of functionally altered neurons neurologically connected with dead and/or dedifferentiated cells. Hatazawa et al. [25] reported a more detailed regional analysis concerning almost the same population studied by Dalakas, also including the evaluation of the motor-sensory cortex at higher levels than used earlier. A brain size correction was added to avoid differences in measured activity depending on brain size, but not from hypometabolism. In this more detailed analysis, a generalized reduction of FDG's uptake was shown in patients with both upper and lower motor neuron disease that was greatest in the motor-sensory cortex and putamen. The motor-sensory deficit was strongly correlated with length of disease, and a marked sequential reduction was seen in the four patients who repeated a PET study. In this paper, a right-left asymmetry in the population described above and a normal or near normal FDG uptake in the four ALS patients without upper motor neuron involvement were also reported. In 2007, the correlation of the extent of cortical lesions with the intensity of motor dysfunction in ALS patients, measured by the ALS functional rating scale score (ALSFRS), has been also studied by Habert et al. [33], who evaluated cerebral perfusion using SPECT with 99mTc-ECD and a statistical parametric mapping (SPM) method. A positive correlation between the degree of involvement of the motor functions and the perfusion decrease of the cerebral cortex was demonstrated. Analyzing the ALSFRS subscores, the cortical involvement was important for lower limbs score, moderate for bulbar score, and below the level of statistical significance for the respiratory and upper limb scores. An asymmetric hypoperfusion, because of a major involvement of the right hemisphere, was seen mainly in the lateral premotor cortex, the insula, and the cingulate cortex.

## 5. Different Patterns of Glucose Metabolism in ALS Patients

Using PET-FDG, different patterns of hypometabolism have been observed when the motor neuron disease (MND) coexists with Frontotemporal Dementia (FTD). Comparing patients with FTD and MND with subjects affected with FTD alone Jeong et al. [34] demonstrated that the patients with FTD/MND showed glucose hypometabolism only in the frontal area, whereas most patients with FTD had hypometabolism in the frontal and temporal areas. Furthermore, in case of FTD/MND, a more symmetric pattern of hypometabolism with respect to patients with FTD alone was showed. To better understand the FDG distribution in patients with FTD, Jeong performed also an SPM analysis in comparison with normal controls [35]. A significant hypometabolism was identified in extensive prefrontal areas, cingulate gyri, anterior temporal regions, and the left inferior parietal lobule and less relevant in the bilateral insula and uncus, left putamen and globus pallidus, and medial thalamic structures. Frontal hypometabolism was more frequently prominent in the left hemisphere than in the right.

Cistaro et al. [36] studied with FDG PET 32 patients with ALS, of either bulbar (*n* = 13) or spinal (*n* = 19) onset, compared by SPM with 22 subjects taken as controls. Patients with spinal onset had significantly higher scores in a neuropsychological test assessing verbal fluency compared with patients with bulbar onset. In this study an unprecedented evidence of relatively increased metabolism in the amygdalae, midbrain, and pons was observed in ALS patients as compared with control subjects, possibly due to local activation of astrocytes and microglia. Highly significant relative decreases in metabolism were found in large frontal and parietal regions in the bulbar onset patients as compared with the spinal onset subjects and the controls, suggesting a differential metabolic and neuropsychological state between the two conditions.

Data of Cistaro et al. [36] are in agreement with an increased metabolism along the course of the cerebral spinal fluid (CSF), at level of pons and midbrain. The hypermetabolism could depend on a colonization of the pyramidal tract by active astrocytes and/or microglia. Interestingly a reduced fractional anisotropy has been observed in the pons and along the CSF in patients who underwent either PET or fMRI [37]. With respect to the hypometabolism observed in patients with a bulbar onset, our results support the presence of an extramotory involvement in ALS which may interest dorsolateral and prefrontal cortex. Conversely, a normal or near normal frontoparietal FDG uptake has been observed in ALS patients with a spinal onset.

In 2014, Cistaro et al. performed another study, valuating the FDG PET profile of 15 patients with familial ALS carrying the GGGGCC hexanucleotide repeat expansion in the C9ORF72 gene and comparing them with a group of 12 patients with ALS and comorbid frontotemporal dementia (FTD) without the C9ORF72 expansion (ALS-FTD), a group of 30 cognitively normal patients and 40 normal controls. The authors demonstrated that, among the 4 groups, patients carrying the C9ORF72 mutation show a more extensive involvement of the central nervous system, with significant hypometabolism in the anterior and posterior cingulate cortex, insula, caudate, and thalamus, the left frontal and superior temporal cortex, and hypermetabolism in the midbrain, bilateral occipital cortex, globus pallidus,, and left inferior temporal cortex [38].

## 6. Microglia Involvement in ALS Patients

The explanation of subcortical hypermetabolic areas as a possible consequence of a colonization by active astrocytes and/or microglia is in agreement with the new view of FDG's cerebral uptake, which changed in the last decades [39]. It has been demonstrated that glucose is not consumed exclusively by neurons and that glucose consumption does not directly reflect only neural activity [40]. In this respect, the importance of astrocytes in glutamate-driven glucose metabolism and regulation has been highlighted [40], supporting the indication that also cells other than neurons are involved in FDG's uptake.

The evidence of widespread cerebral microglial activation in amyotrophic lateral sclerosis may be further understood reading the paper by Turner et al. [41], who reported an experience with [11C](R)-PK11195, a ligand for the peripheral benzodiazepine binding site, expressed by activated microglia. The PET study has been performed in ten ALS patients and 14 healthy controls. Significantly increased binding was found in motor cortex, pons, dorsolateral prefrontal cortex, and thalamus in the ALS patients, with significant correlation between binding in the motor cortex and the burden of upper motor neuron signs clinically evident. This paper supports the interest for the development of therapeutic strategies in ALS aimed at inflammatory pathways. Favourable results have already been obtained in experimental models where an increased survival has been observed in animals when treated with anti-inflammatory drugs. To demonstrate the relevance of astrocytosis in ALS, Johansson et al. [42] utilized [11C](L)-deprenyl-D2, which binds to the enzyme MAO-B, primarily located in astrocytes. An increased uptake of deuterium-substituted [11C](L)-deprenyl PET was demonstrated in pons and white matter of seven patients with ALS, compared with seven healthy control subjects.

## 7. Evaluation of ALS Patients with PET Receptor Studies

An original study has been published by Lloyd et al. [43], who evaluated extramotor involvement in ALS, using as PET radiotracer the [(11)C]flumazenil (FMZ), a benzodiazepine GABA(A) marker. The study was performed in seventeen nondemented patients with clinically definite or probable ALS compared with seventeen normal controls. The analysis was based on SPM maps, derived to localize changes in regional flumazenil volumes of distribution (FMZVD), which correlate closely with receptor density. Relative FMZVD was significantly decreased in the ALS group in the prefrontal cortex, parietal cortex, visual association cortex, and left motor/premotor cortex. A relative reduction in FMZVD was also present, though less evident, in the left ventrolateral and dorsolateral prefrontal cortex, Broca's area, and the right temporal and right visual association cortex. These data are in agreement with a cerebral dysfunction in ALS which involves not only the motor cortex, but also premotor and extramotor areas, particularly in the prefrontal regions. A more recent study using [11C]flumazenil (FMZ) PET has been performed by Turner et al. [44], who focalized their interest in patients with ALS, also including subjects who presented the “D90A” SOD1 mutation. The mutations of the superoxide dismutase-1 (SOD1) gene are associated with five to ten percent cases of ALS. Between them, the “D90A” mutation individuate a unique phenotype, characterized by a markedly slower disease progression, with a mean survival of 14 years, probably dependent on the relative sparing of inhibitory cortical neuronal circuits. The study has been based on the comparison of results obtained in twenty-four sporadic ALS (sALS), 10 homozygous D90A patients, and two subjects homozygous for the D90A mutation, but without symptoms or signs (“presymptomatic”, psD90A), with those achieved in 24 age-matched normal controls. While in sALS a decreased uptake has been observed within premotor regions, motor cortex, and posterior motor association areas; in the homD90A group the reduction was concentrated in the left frontotemporal junction and anterior cingulate gyrus. In the two psD90A subjects, a small focus of reduced uptake was seen at the left frontotemporal junction, therefore showing a pattern similar to the one observed in the clinically affected patients. No statistically significant association between the reduction in cortical FMZ binding and revised Amyotrophic Lateral Sclerosis Functional Rating Scale (ALSFRS) was demonstrated in sALS patients, whereas the upper motor neuron (UMN) score correlated with widespread and marked cortical decreases over the dominant hemisphere. Conversely, in the D90A group, the decreased in FMZ uptake was strongly statistically associated with ALSFRS-R, rather than the UMN score, being also related to the disease's duration.

To evaluate the possible interest of dopaminergic radiotracers in ALS, Takahashi et al. [45] utilized F-18 fluorodopa in 16 patients with sporadic ALS and without extrapyramidal disease, compared with age-matched controls. A significant progressive fall in fluorodopa uptake was observed in 3 patients with ALS of long duration.

The possible interest of radiotracers of serotonin was inquired by Turner et al. [41], who performed PET with [11C]-WAY100635 PET, a sensitive marker of in vivo 5-HT1A receptor binding, in ALS patients compared with controls. An SPM analysis evidenced a striking and widespread decrease in cerebral 5-HT1A binding in ALS patients compared with controls in both motor and extramotor regions, with the most marked changes in frontotemporal regions. Their hypothesis was that these findings reflect widespread damage to cortical pyramidal neurones that express 5-HT1A receptors, although a purely functional change in receptor binding cannot be excluded.

## 8. Conclusion

In the older definition ALS is considered as a disease exclusively characterized by preferential loss of motor neurons in the motor cortex, brainstem, and spinal cord, that is, by a pathological involvement exclusively of the motor system. Although they do not have yet been included in the clinical scenario, PET and SPECT may give interesting information in these patients, having capability to trace many important pathophysiological and biochemical targets involved in the disease. The greatest advantage achievable by molecular imaging is in individuating early functional alterations, preceding the morphostructural evidence, better explaining clinical symptoms, as those connected with a frontal dementia, and helping to define a prognostic stratification. At the present, clinical information may be mainly acquired using PET-FDG. Important data, to be utilized as premise to a therapeutic strategy based on inflammation as a target, could be achieved with radiotracers allowing to detect astrocytosis. Finally, having been demonstrated a possible role of fMRI in detecting alterations in fractional anisotropy, at level of subcortical structures, very intriguing perspectives could be associated with the diffusion of PET-MRI hybrid machines, allowing obtaining simultaneously, together with the morphostructural information, functional data acquirable either with PET or fMRI.

## Figures and Tables

**Table 1:** ALS diagnostic categories according to the El Escorial World Federation of Neurology diagnostic criteria.

Clinically definite ALS	Defined on clinical evidence alone by the presence of UMN, as well as LMN signs, in three regions

Clinically probable ALS	Defined on clinical evidence alone by UMN and LMN signs in at least two regions with some UMN signs necessarily rostral to (above) the LMN signs.

Clinically probable-laboratory-supported ALS	Defined when clinical signs of UMN and LMN dysfunction are in only one region, or when UMN signs alone are present in one region, and LMN signs defined by EMG criteria are present in at least two limbs, with proper application of neuroimaging and clinical laboratory protocols to exclude other causes.

Clinically possible ALS	Defined when clinical signs of UMN and LMN dysfunction are found together in only one region or UMN signs are found alone in two or more regions; or LMN signs are found rostral to UMN signs and the diagnosis of clinically probable-laboratory-supported ALS cannot be proven by evidence on clinical grounds in conjunction with electrodiagnostic, neurophysiologic, neuroimaging, or clinical laboratory studies. Other diagnoses must have been excluded to accept a diagnosis of clinically possible ALS
